# Nitric oxide is involved in the oxytetracycline-induced suppression of root growth through inhibiting hydrogen peroxide accumulation in the root meristem

**DOI:** 10.1038/srep43096

**Published:** 2017-02-21

**Authors:** Qing-Xiang Yu, Golam Jalal Ahammed, Yan-Hong Zhou, Kai Shi, Jie Zhou, Yunlong Yu, Jing-Quan Yu, Xiao-Jian Xia

**Affiliations:** 1 Department of Horticulture, Zijingang Campus, Zhejiang University, Hangzhou 310058, China; 2 Institute of Pesticide & Environmental Toxicology, Zijingang Campus, Zhejiang University, Hangzhou 310058, China; 3 Zhejiang Provincial Key Laboratory of Horticultural Plant Integrative Biology, Hangzhou 310058, China

## Abstract

Use of antibiotic-contaminated manure in crop production poses a severe threat to soil and plant health. However, few studies have studied the mechanism by which plant development is affected by antibiotics. Here, we used microscopy, flow cytometry, gene expression analysis and fluorescent dyes to study the effects of oxytetracycline (OTC), a widely used antibiotic in agriculture, on root meristem activity and the accumulation of hydrogen peroxide (H_2_O_2_) and nitric oxide (NO) in the root tips of tomato seedlings. We found that OTC caused cell cycle arrest, decreased the size of root meristem and inhibited root growth. Interestingly, the inhibition of root growth by OTC was associated with a decline in H_2_O_2_ levels but an increase in NO levels in the root tips. Diphenyliodonium (DPI), an inhibitor of H_2_O_2_ production, showed similar effects on root growth as those of OTC. However, exogenous H_2_O_2_ partially reversed the effects on the cell cycle, meristem size and root growth. Importantly, cPTIO (the NO scavenger) and tungstate (an inhibitor of nitrate reductase) significantly increased H_2_O_2_ levels in the root tips and reversed the inhibition of root growth by OTC. Out results suggest that OTC-induced NO production inhibits H_2_O_2_ accumulation in the root tips, thus leading to cell cycle arrest and suppression of root growth.

Soil organic matter (SOM), the so-called “soul of soil,” is gradually diminished, owing to intensive vegetable cultivation in greenhouses. To supplement incompletely replenished levels of SOM, animal manure and other organic fertilizers are often applied to the soil and increase not only total soil nutrient content and the activity of soil microorganisms but also soil aggregation, macropore volume, saturated hydraulic conductivity, and soil water-holding capacity, thus improving soil physical, chemical and microbial properties^1^,^2^,^3^. However, the modern livestock industry uses enormous amounts of antibiotics for the prevention and control of diseases. Antibiotics cannot be completely absorbed or metabolized by animals, and eventually 30–90% of antibiotics are released into the environment via animal excreta, which is commonly used as manure^4^,^5^. It has been reported that concentrations of tetracycline and chlortetracycline can reach 4.0 and 0.1 mg/kg, respectively, in manure^6^. Therefore, extensive use of antibiotic-containing manure poses a severe threat to environmental health.

In addition to the development of antibiotic resistance in naturally occurring pathogens, accumulation of antibiotics in crop plants and subsequent contamination of the food chain can potentially damage human health^7^. To date, the ecological toxicity of antibiotic contamination is an urgent issue in environmental science. Several studies have clearly demonstrated that the phytotoxicity of antibiotics have detrimental effects on crop yield. For instance, high concentrations of antibiotics such as chlortetracycline and oxytetracycline adversely affect seed germination, root growth and the early development of seedlings in a range of plant species^8^,^9^,^10^. Furthermore, exposure of maize plants to chlortetracycline induces glutathione *S*-transferase (GST) and peroxidase, both of which play critical roles in transforming chlortetracycline into less toxic glutathione conjugates^11^. More recently, it has been demonstrated that cucurbit crops exposed to active pharmaceutical ingredients exhibit growth retardation due to disturbed nutrient and hormone homeostasis^12^. Although these studies have revealed plant responses to high concentrations of antibiotics, studies demonstrating plant developmental responses at both the cellular and molecular levels to lower concentrations of antibiotics are still scarce.

The organized temporal and spatial distribution of different cell types in plant roots makes roots an ideal model to study plant development. Plant root comprises the meristem zone where cell division occurs and the elongation zone where cells undergo differentiation while cell size rapidly increases^13^. During seed germination and early development, the meristem activity of roots is sensitive to drought, high salinity, and heavy metals in the soil, all of which cause oxidative stress^14^,^15^,^16^. Previous studies have shown that oxidative stress induces a checkpoint pathway that modulates cyclin genes^17^. In a broad sense, cyclins and cyclin-dependent kinases (CDKs) form protein complexes and control the plant cell cycle. Different CDK-cyclin complexes activate substrates at key G1-to-S and G2-to-M transition points, triggering the onset of DNA replication and mitosis, respectively^18^. Therefore, the question of how cell division in the root meristem zone is affected by antibiotics is fundamental to understanding the mechanism of developmental defects in antibiotic-contaminated soil. In this study, we analyzed the effects of oxytetracycline (OTC), a widely used antibiotic in agriculture, on root growth during the early development of tomato seedlings, with a focus on cell division status and the expression of cell-cycle genes in the root tips. Importantly, the accumulation of hydrogen peroxide (H_2_O_2_) and nitric oxide (NO) in the root tips and their roles in OTC-induced suppression of root growth were determined. Our results indicate that OTC has detrimental effects on the cell cycle in the root meristem and on root growth, whereas OTC-induced NO plays a role in inhibiting root meristem activity by inhibiting H_2_O_2_ accumulation.

## Results

### Oxytetracycline inhibited the root growth of tomato seedlings

**Figure 1:**
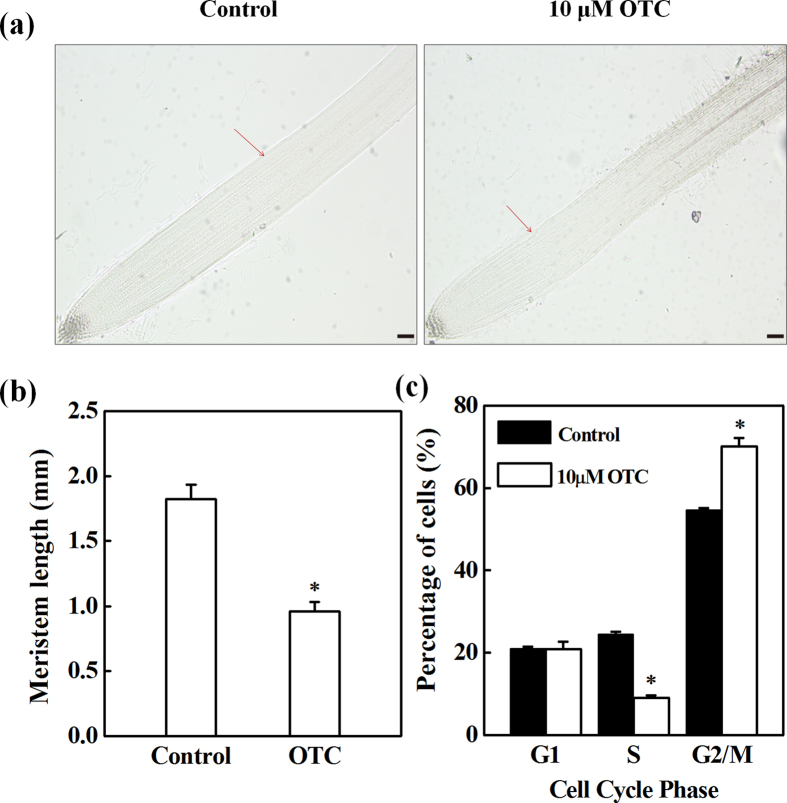
Inhibition of cell division by oxytetracycline (OTC) in tomato root tips. (**a**,**b**) Effects of 10 μM OTC on root meristem size in tomato seedlings. Arrows indicate the boundary between the meristem and the elongation zone of the root. (**c**) Percentage of cell numbers in different phases of the cell cycle in the root tips treated with or without 10 μM OTC. Each sample contains at least 50 root tips. Data represent mean ± SE (n = 5, *P value < 0.05, two-sided Student’s t test). The bar indicates 120 μm.

To study the effects of oxytetracycline (OTC) on root growth during the early development of tomato seedlings, we analyzed the root growth of seedlings grown in MS medium with varying concentrations of OTC. OTC at 10 μM was found to significantly inhibit primary root growth and lateral root development (Fig. S1). Thus, 10 μM OTC was used in the following experiments. Microscopic observation showed that inhibition of root growth was associated with a decreased root meristem size in OTC-treated seedlings (Fig. 1a,b). Furthermore, flow cytometry indicated that the number of S-phase cells, which underwent DNA replication, was significantly inhibited by 63% in the root tips after OTC treatment, whereas the number of G1-phase cells was not affected (Fig. 1c). Accordingly, the number of G2-phase cells was significantly increased by OTC treatment.

### OTC inhibited the cell cycle in the root tips

**Figure 2:**
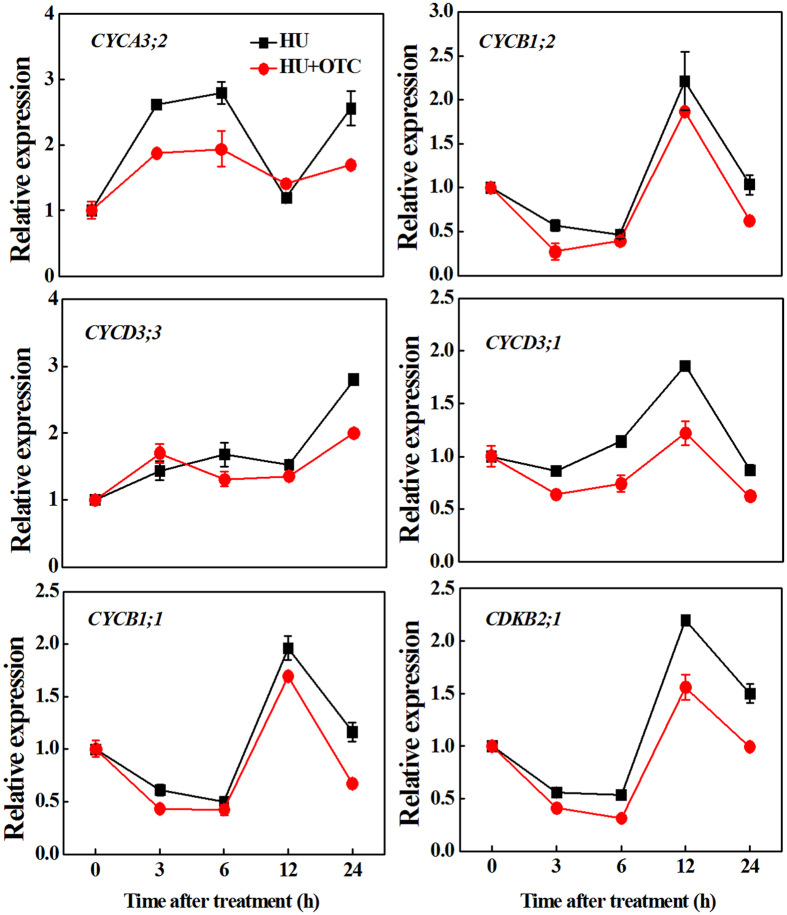
Time-course analysis of expression of cyclin genes (*CYCA3;2, CYCD3;3, CYCB1;1, CYCB1;2, CYCD3;1*) and *CDKB2;1* gene (encoding cyclin-dependent kinase B2;1) in tomato root tips at 0, 3, 6, 12 and 24 h after treatment with 10 μM oxytetracycline (OTC). Cell division in the root tips was synchronized by using 2 mM hydroxyurea (HU).

To further study the mechanism of root growth arrest by OTC, we analyzed cell cycle-related genes in the root tips. The cell cycle was synchronized by addition of hydroxyurea (HU) to the growth medium. The time-course of gene expression showed that the transcript level of *CYCA3;2*, an S-phase gene, increased sharply 3 h after transfer to the HU-containing medium, reaching a maximum at 6 h, and was followed by a decline at 12 h (Fig. 2). However, the expression of *CYCD3;3*, a G1-phase gene, was relatively stable during this period (3–12 h). Interestingly, G2/M marker genes (*CYCB1;1, CYCB1;2, CYCD3;1* and *CDKB2;1*) showed complementary expression patterns, which steadily decreased until 6 h and peaked at 12 h, when S-phase genes declined. As G2/M genes were downregulated at 24 h, *CYCA3;2* and *CYCD3;3* transcripts accumulated again, thus suggesting the onset of a second cell cycle. Importantly, OTC treatment inhibited the upregulation of *CYCA3;2* from 3 to 6 h and the expression peaks of G2/M marker genes at 12 h. In addition, expression levels of all cell cycle genes were inhibited by OTC at 24 h. Together, these results suggested that OTC treatment caused inhibition of the cell cycle in the root tips.

### OTC inhibited H_2_O_2_ accumulation in the root tips

**Figure 3:**
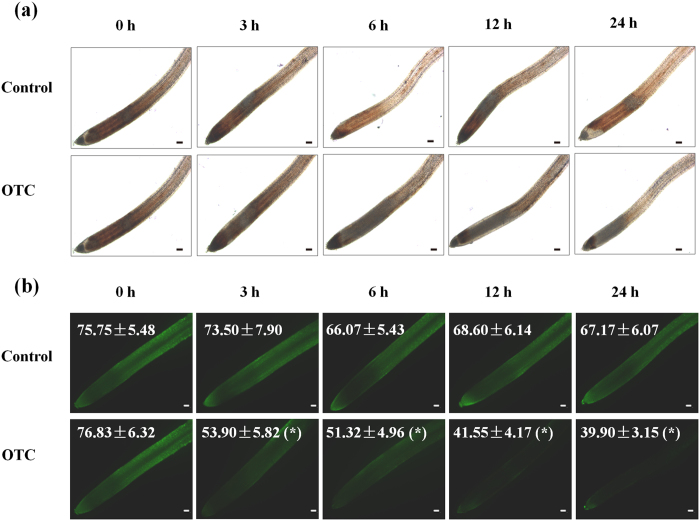
Changes in H_2_O_2_ levels over time in root tips after treatment with 10 μM oxytetracycline (OTC) or no treatment. H_2_O_2_ was visualized by staining with DAB (**a**) or the fluorescent dye H_2_DCF-DA (**b**). Fluorescence intensity for H_2_O_2_ accumulation in the root tips is shown in each image. Data are the means of three replicates with SE (*P value < 0.05, two-sided Student’s t test). The bar indicates 120 μm.

**Figure 4:**
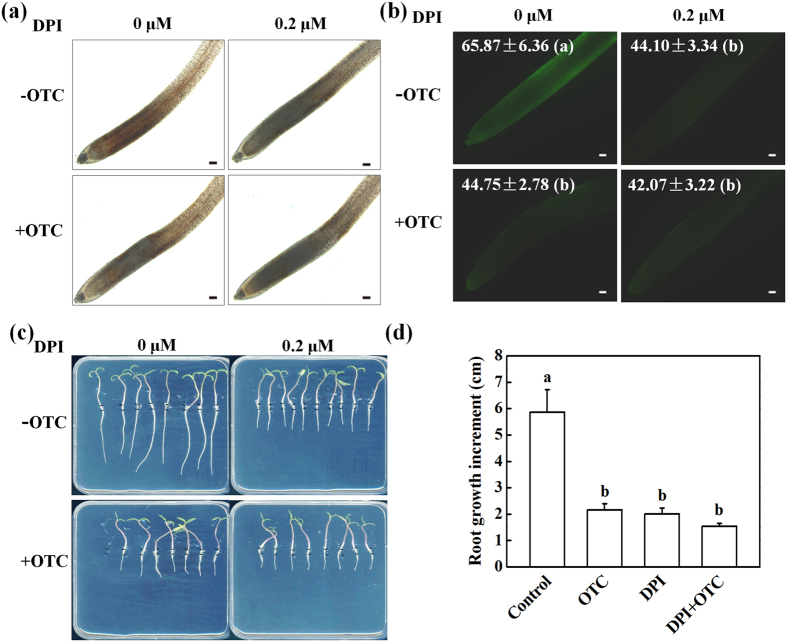
Root growth defects induced by oxytetracycline (OTC) were associated with a decline in H_2_O_2_ levels in root tips. (**a**,**b**) Effects of oxytetracycline (OTC), diphenyliodonium (DPI) or OTC + DPI treatment on H_2_O_2_ levels in tomato root tips. OTC and DPI were applied at 10 μM and 0.2 μM, respectively. H_2_O_2_ was visualized by staining with DAB (**a**) or H_2_DCF-DA (**b**) 24 h after treatments. The fluorescence intensity for H_2_O_2_ accumulation in the root tips is shown in each image. Data are the means of three replicates with SE. The bar indicates 120 μm. (**c**,**d**) Effects of treatment with OTC, DPI or OTC + DPI on primary root growth. Measurements were taken 3 d after treatments, and data represent mean ± SE (n = 15). Means followed by the same letter are not significantly different according to Tukey’s test (*P* < 0.05).

Oxidative stress can be induced by a wide range of organic pollutants and is usually associated with growth inhibition in plants. We analyzed H_2_O_2_ accumulation, as visualized by DAB and DCF staining, which reveal H_2_O_2_ on the basis of red-brown color and fluorescence signal in the root tips, respectively. Interestingly, the levels of H_2_O_2_ in the root tips gradually declined from 3 h after transfer to OTC-containing medium, whereas H_2_O_2_ levels remained stable during that time-course in the control (Fig. 3a,b). Twenty-four hours after OTC treatment, the DCF fluorescence intensity was decreased by 40.6% compared with that in the control (Fig. 3b). To understand the physiological relevance of the decrease in H_2_O_2_ levels in root tips, we compared OTC and DPI, a well-known inhibitor of reactive oxygen species (ROS) production, to determine their effects on H_2_O_2_ accumulation and root growth. Interestingly, 10 μM OTC or 0.2 μM DPI treatment resulted in similar inhibition in H_2_O_2_ accumulation (Fig. 4a,b). OTC or DPI treatment resulted in a 32% or 33% decline in DCF fluorescence intensity (Fig. 4b). Furthermore, OTC or DPI treatment led to a 63% or 65% decrease in root growth (Fig. 4c,d). In addition, combined OTC and DPI treatment did not show significant additive effects on the inhibition of root growth. The results suggested that OTC-induced arrest of root growth may be related to decreased H_2_O_2_ levels in root tips.

### Exogenous H_2_O_2_ partially reversed OTC-induced inhibition of root growth

**Figure 5:**
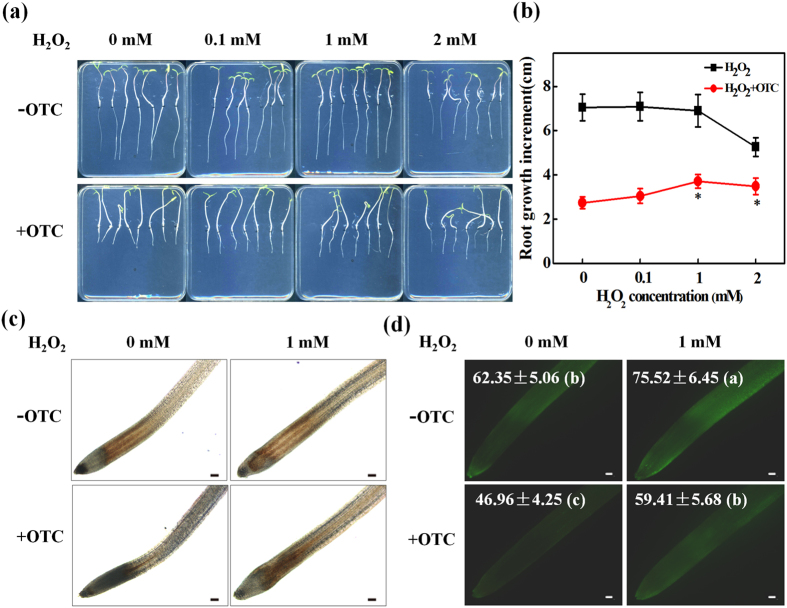
H_2_O_2_ ameliorated the root growth defect induced by oxytetracycline (OTC). Effects of treatment with different concentrations of H_2_O_2_ on primary root growth (**a**,**b**) and H_2_O_2_ levels in tomato root tips (**c**,**d**) in the presence or absence of 10 μM OTC. H_2_O_2_ was visualized by staining with DAB (**c**) or H_2_DCF-DA (**d**). The fluorescence intensity for H_2_O_2_ accumulation in the root tips is shown in each image. The bar indicates 120 μm. For root length, data represent mean ± SE (n = 15, *P value < 0.05, two-sided Student’s t test). For fluorescence intensity, data are the means of three replicates with SE. Means followed by the same letter are not significantly different according to Tukey’s test (*P* < 0.05).

**Figure 6:**
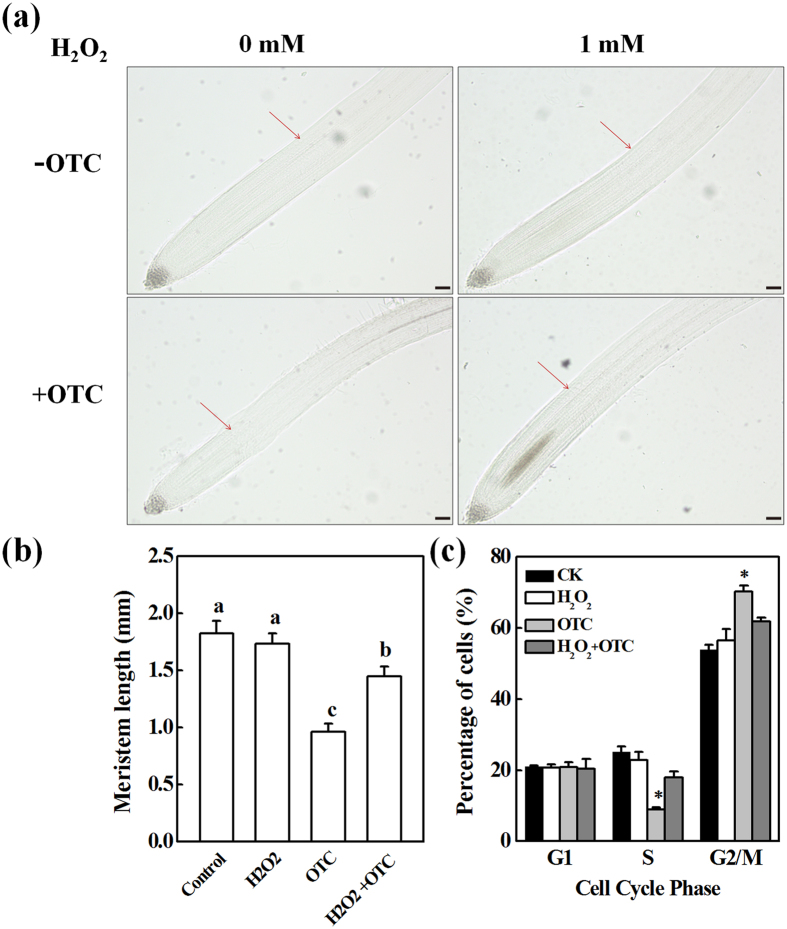
H_2_O_2_ ameliorated inhibition of cell division by oxytetracycline (OTC) in tomato root tips. (**a**,**b**) Effect of 1 mM H_2_O_2_ on the size of root meristem in tomato seedlings with or without 10 μM OTC treatment. Arrows indicate the boundary between the meristem and the elongation zone of the root. The bar indicates 120 μm. Data represent mean ± SE (n = 15). Means followed by the same letter are not significantly different according to Tukey’s test (*P* < 0.05). (**c**) Percentage of cell numbers in different cell-cycle phases in the root tips. Each sample contains at least 50 root tips. Data represent mean ± SE (n = 5, *P value < 0.05, two-sided Student’s t test).

To further understand the relationship between the OTC-induced decline in H_2_O_2_ levels in root tips and inhibition of root growth, varying concentrations of H_2_O_2_ were added to the growth medium with or without OTC, and the root growth was analyzed. H_2_O_2_ treatment alone inhibited root growth at 2 mM (Fig. 5a,b). However, H_2_O_2_ at 1 mM did not have significant effects on root growth and partially recovered the inhibition of root growth by OTC. DAB and DCF staining confirmed that 1 mM H_2_O_2_ application significantly increased H_2_O_2_ levels in the root tips after OTC treatment (Fig. 5c,d). The DCF fluorescence intensity of OTC-exposed roots increased by 26.5% after H_2_O_2_ treatment (Fig. 5d). Moreover, recovery of root growth from the inhibition by OTC was associated with an increase in root meristem size after H_2_O_2_ treatment (Fig. 6a,b). Accordingly, the number of S-phase cells in the root tips significantly increased (1.9-fold), whereas the number of G2/M cells decreased (Fig. 6c). The results confirmed that OTC inhibited the cell cycle and root growth through decreased H_2_O_2_ levels in the root tips.

### Inhibition of root growth by OTC was associated with nitric oxide production

**Figure 7:**
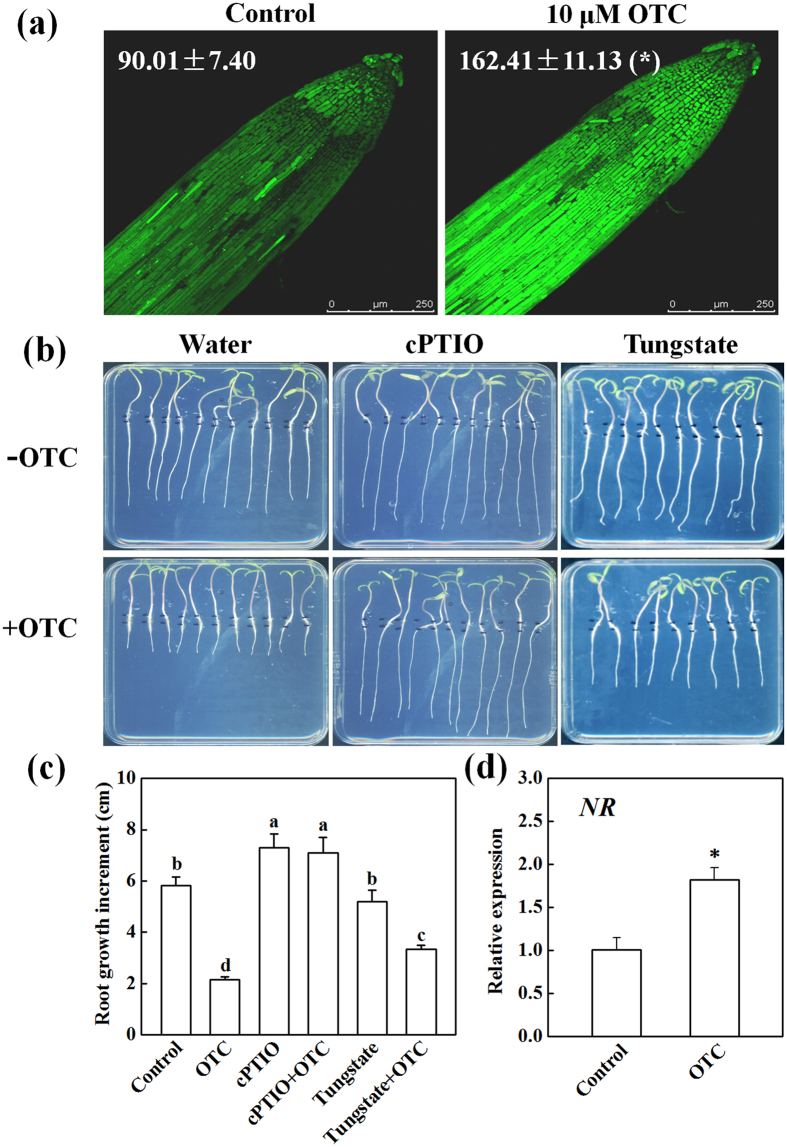
Nitric oxide (NO) was involved in oxytetracycline (OTC)-induced inhibition of tomato root growth. (**a**) Detection of NO by staining with fluorescent dye DAF-FM DA at 24 h after OTC treatment. The fluorescence intensity for NO accumulation in the root tips is shown in each image. Data are the means of three replicates with SE (*P value < 0.05, two-sided Student’s t test). (**b**,**c**) Effects of 250 μM cPTIO (the NO scavenger), and 100 μM tungstate (the inhibitor of nitrate reductase, NR) on root growth of tomato seedlings in the presence or absence of 10 μM OTC. Measurements were taken 3 d after treatments, and data represent mean ± SE (n = 15). Means followed by the same letter are not significantly different according to Tukey’s test (*P* < 0.05). (**d**) Effect of treatment with OTC on expression of *NR* gene in the root tips. Samples were collected 24 h after treatment. Data represent mean ± SE (n = 5, *P value < 0.05, two-sided Student’s t test).

Plant responses to stress are often modulated by interactions between ROS and nitric oxide (NO). To study the involvement of NO in OTC-induced arrest of root growth, we analyzed NO accumulation in the root tips. In contrast to H_2_O_2_, OTC significantly increased NO levels in the root tips (Fig. 7a). In addition, OTC treatment induced upregulation of the *NR* gene (encoding nitrate reductase, NR) in the root tips (Fig. 7d). Interestingly, the inhibition of root growth by OTC was completely and partially recovered by cPTIO (the specific NO scavenger) and tungstate (an inhibitor of NR), respectively (Fig. 7b,c). However, tungstate alone did not affect root growth, and cPTIO alone only slightly promoted root growth.

**Figure 8:**
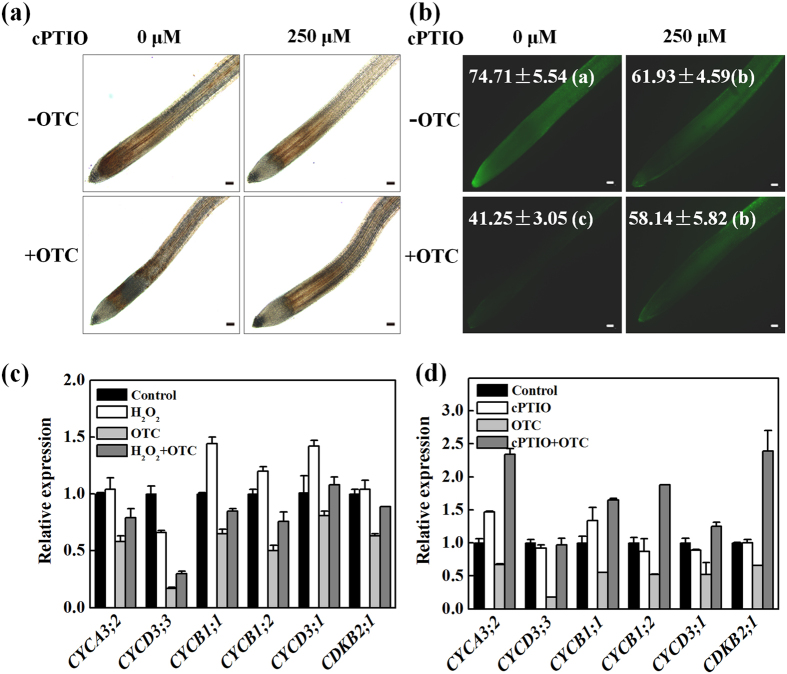
Nitric oxide (NO) was involved in oxytetracycline (OTC)-induced decline in H_2_O_2_ levels and arrest of the cell cycle in tomato root tips. (**a**,**b**) Effects of 250 μM cPTIO, scavenger of NO on H_2_O_2_ levels and expression of cell cycle genes in tomato root tips in the presence or absence of 10 μM oxytetracycline (OTC). H_2_O_2_ was visualized by staining with DAB (**a**) or H_2_DCF-DA (**b**). The fluorescence intensity for H_2_O_2_ accumulation in the root tips is shown in each image. Measurements were taken 24 h after treatments, and data are the means of 3 replicates with SE. Means followed by the same letter are not significantly different according to Tukey’s test (*P* < 0.05). The bar indicates 120 μm. (**c**,**d**) Effects of 250 μM cPTIO and 1 mM H_2_O_2_ on the expression of cyclin genes (*CYCA3;2, CYCD3;3, CYCB1;1, CYCB1;2, CYCD3;1*) and *CDKB2;1* gene (encoding cyclin-dependent kinase B2;1) in tomato root tips in the presence or absence of OTC. Samples were collected 24 h after treatment.

In parallel to the recovery of root growth, cPTIO significantly increased H_2_O_2_ levels in the root tips, as shown by DAB and DCF staining after OTC treatment (Fig. 8a,b). The DCF fluorescence intensity of OTC-exposed roots increased by 40.9% after cPTIO treatment (Fig. 8b). However, cPTIO alone had no effects on H_2_O_2_ levels in the root tips. To confirm the roles of H_2_O_2_ and NO on root growth in response to OTC, we analyzed the effects of exogenous application of H_2_O_2_ or cPTIO on the expression of cell cycle genes. OTC significantly inhibited expression of selected genes 24 h after treatment (Fig. 8c,d). Interestingly, H_2_O_2_ (partially) and cPTIO (completely) recovered the inhibition of gene expression by OTC. However, H_2_O_2_ or cPTIO alone had almost no effects on the expression of cell cycle genes, except that H_2_O_2_ alone inhibited the expression of *CYCD3;3*.

## Discussion

Primary root growth is determined by the rate of cell division in the apical meristem and cell elongation in the elongation zone^13^. We found that OTC treatment strongly inhibited root growth, which in turn was associated with decreased root meristem size (Fig. 1). This finding suggests that OTC suppressed root growth by inhibiting the mitotic activity of the root apical meristem. Downregulation of cell cycle genes further indicated that OTC inhibited cell division in the root meristem (Figs 2 and 8). In agreement with results from previous studies, the cell cycle genes showed a specific expression rhythm in the presence of HU, which synchronizes cell cycles without causing significant negative effects on root growth^19^. The *CYCA3;2* transcript level, which was high from 3–6 h, was downregulated at 12 h (Fig. 2). In contrast, transcript levels of *CYCB1;1, CYCB1;2, CYCD3;1* and *CDKB2;1* were low from 3–6 h. However, the genes were activated at 12 h. The temporal transcriptional patterns suggested roles for *CYCA3;2* as an S-phase gene and *CYCB1;1, CYCB1;2, CYCD3;1* and *CDKB2; 1* as G2/M genes^20^. Previous studies have shown that oxidative stress inhibits the expression of cell cycle genes and impairs the G1/S transition, slows DNA replication and delays entry into mitosis^17^. Similarly, OTC inhibited the upregulation of *CYCA3;2* during S-phase when DNA replication occurred and inhibited the expression peak of G2/M genes at 12 h (Fig. 2). Accordingly, OTC significantly decreased the number of S-phase cells but resulted in a higher number of G2 cells in the root tips (Fig. 1c). Therefore, it is likely that OTC inhibited both the G1-S and G2-M transitions in the cell cycle. However, OTC significantly decreased H_2_O_2_ accumulation in the root tips (Fig. 3). Notably, OTC had similar effects on root growth as DPI, an inhibitor of ROS production; combined treatment with OTC and DPI did not show additive effects on root growth (Fig. 4c,d). This result suggests that an H_2_O_2_-dependent pathway is a common target of OTC and DPI. In fact, dynamic and robust control of ROS levels is critical for plant growth regulation^21^. Gibberellin (GA)-regulated seed germination is associated with spatial and temporal changes in ROS^22^, whereas DELLA protein, a negative regulator of GA signaling, decreases ROS levels and inhibits both cell elongation and cell proliferation in roots^23^,^24^. In addition, UPBEAT1, a transcription factor suppressing the expression of peroxidases, regulates superoxide radicals and H_2_O_2_ distribution and controls the balance between cell proliferation and differentiation in roots^25^. Our results, together with those from previous studies, demonstrate that cell cycle arrest by OTC is attributable to decreased H_2_O_2_ levels. Because ROS production is an intrinsic part of auxin signaling, which is critical for maintenance of root meristem activity^26^, further studies should investigate whether OTC affects auxin levels and distribution in root tips.

In contrast to H_2_O_2_, the accumulation of NO was significantly induced by OTC in the root tips (Fig. 7a). Suppression of NO accumulation by both cPTIO and tungstate ameliorated OTC-induced growth inhibition (Fig. 7b,c), thus suggesting that OTC-induced NO production is detrimental to root growth. Notably, cPTIO almost completely recovered OTC-induced root growth arrest (Fig. 7b,c). This result suggests that NO may be the primary cause of OTC-induced cell cycle arrest and inhibition of root growth. NO has been shown to cause root apical meristem defects and growth inhibition by interfering with auxin polar transport^27^. In addition, excessive NO may decrease cytokinin levels and/or inhibit cytokinin signaling, whereas cytokinin regulates cell division and plant growth through the D-type cyclin CYCD3;1^28^,^29^,^30^,^31^. It is likely that OTC-induced NO production causes hormonal imbalances in root meristem.

Of particular interest, cPTIO reversed the inhibition of H_2_O_2_ accumulation by OTC (Fig. 8a,b), thus suggesting that OTC-induced NO may inhibit the accumulation of H_2_O_2_ in root meristem and consequently lead to an arrest of the cell cycle and root growth. Previously, several studies have reported contrasting conclusions regarding the relationship between NO and H_2_O_2_ in plant responses to stress. NR-dependent NO acts downstream of RBOH-mediated H_2_O_2_ in abscisic acid-induced stomatal closure in Arabidopsis^32^. However, NO has been shown to positively regulate H_2_O_2_ levels in UV-B-induced stomatal closure in broad bean^33^. In addition, H_2_O_2_ and NO synergistically trigger plant cell death^34^. Therefore, the relationship between H_2_O_2_ and NO varies, depending on plant species, treatments and/or experimental systems. Interestingly, depletion of NO results in elevated reactive oxygen species levels, abnormal root meristem and perturbed auxin signaling in Arabidopsis^35^. This result further supports our finding that OTC-induced NO decreased H_2_O_2_ levels in root meristem (Fig. 8b) and indicated that crosstalk between H_2_O_2_ and NO in a developmental context appears to differ from that in plant responses to stress. Interestingly, lower levels of NO enhance rootward auxin transport, whereas high levels of NO decrease rootward auxin transport^35^. Auxin activates RAC/ROP small GTPases, which regulate the ROS-producing activity of NADPH oxidase^36^, whereas an auxin-regulated oxidizing environment is critical for maintenance of the root meristem^26^. Suppressed auxin signaling due to OTC-induced NO production may lead to a decline in H_2_O_2_ levels (Fig. 8b), possibly as a result of dampened ROP activity. In this case, crosstalk between ROS and auxin may be disrupted by OTC, thus leading to arrest of the cell cycle and root growth.

Although OTC-induced arrest of the cell cycle and root growth is related to redox imbalance, the mechanism by which OTC induces NO production is still unknown. The OTC-induced inhibition of root growth was ameliorated by an NR inhibitor (Fig. 7b,c). Moreover, OTC treatment induced the upregulation of the *NR* gene (Fig. 7d). These results suggested that NR may be a major source of NO production after OTC treatment. We also analyzed the effects of different concentrations of L-NAME, an inhibitor of nitric oxide synthase (NOS) in mammals, on root growth in the presence or absence of OTC (Fig. S2). However, L-NAME did not ameliorate root growth inhibition by OTC. In fact, the true NOS homologs in plants have not yet been identified^37^. Thus, we propose that OTC probably induces NO via NR. We also observed chlorosis in newly emerged leaves when roots were treated with OTC in hydroponically grown tomato plants (data not shown). This finding indicates that nitrogen metabolism may be targeted by OTC in tomato plants. OTC has long been known to inhibit protein synthesis by binding to ribosome in bacteria^38^. Whether OTC exerts similar properties in plant cells is unknown. However, the plant plastidial GTPase NOA1, which regulates NO production, shows homology to members of the YlqF/YawG family of P-loop GTP binding proteins, which play roles in ribosomal biogenesis and protein translation^39^. Thus, there is probably an intrinsic relationship between protein biosynthesis and NO production. Whether OTC regulates NO production by interrupting ribosome assembly via direct or indirect interaction with NOA1 will require further study.

**Figure 9:**
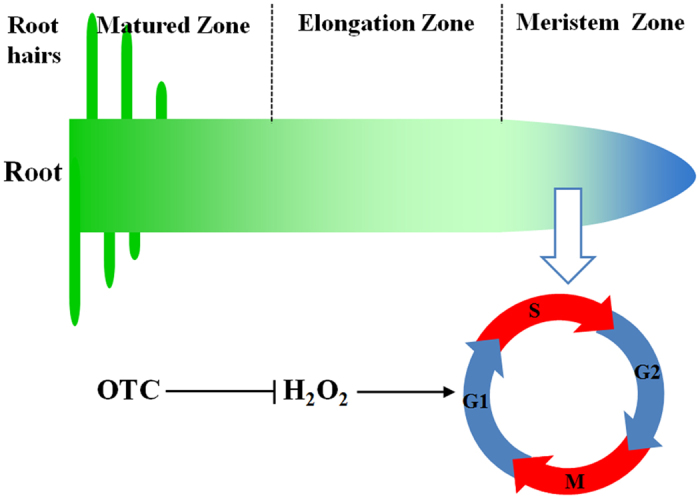
Model of the mechanism of inhibition of root growth by oxytetracycline (OTC) in tomato seedlings. OTC treatment results in a decline in H_2_O_2_ levels in root tips. H_2_O_2_ is a critical signal for maintaining the cell cycle in root meristem. Low H_2_O_2_ levels in the root tips as a result of OTC treatment lead to decreased root meristem size and root growth.

In conclusion, OTC decreases meristem size and overall root growth by inhibiting the cell cycle. OTC inhibited the accumulation of H_2_O_2_ in the root tips, which play positive roles in the cell cycle and root growth (Fig. 9). It is clear that soil OTC contamination can be detrimental to plant growth. To fulfill the demand of phytoremediation of tetracycline-type antibiotics, it is critical to increase root tolerance, especially during the early development of seedlings. Maintaining root redox balance is particularly important in the improvement of root growth in antibiotic-contaminated soil. However, control of redox and hormonal homeostasis of roots in polluted soil is quite challenging. Aboveground stimuli, such as foliar application of brassinosteroid, that trigger ROS production in roots through long-distance signaling are expected to be an effective approach.

## Methods

### Chemical reagents

Oxytetracycline (OTC), diphenyleneiodonium (DPI), 2-phenyl-4,4,5,5- tetramethylimidazoline-1-oxyl-3-oxide (cPTIO) and N^G^-nitro-L-Arg methyl ester (L-NAME) used for seedling treatments were purchased from Sigma-Aldrich (Sigma-Aldrich, St. Louis, MO, USA). The fluorescent dyes 2′,7′-dichlorodihydrofluorescein diacetate (H_2_DCF-DA) and 4-amino-5-methyl-amino-2′,7′-difluorofluorescein diacetate (DAF-FM DA) used for staining of H_2_O_2_ and nitric oxide, respectively, were obtained from Molecular Probes (Thermo Fisher Scientific, Waltham, MA USA), and 3,3-diaminobenzidine (DAB) was obtained from Sigma-Aldrich (Sigma-Aldrich, St. Louis, MO, USA). Hydrogen peroxide (H_2_O_2_) was purchased from Dingguo Biotech Co., Ltd (Shanghai, China).

### Treatments and growth conditions

Tomato seeds (*Solanum lycopersicum* L. cv. Hezuo 903) were surface-sterilized with 75% ethanol for 30 s and 10% NaClO for 15 min and rinsed five times with double-distilled water (ddH_2_O). Seeds were gently shaken for 6 h and washed with ddH_2_O five times. Sterilized seeds were placed on half-strength MS basal medium (pH5.8), containing 1.5% sucrose and 0.8% agar. Seeds were germinated on vertically placed 13 cm × 13 cm petri plates in a growth chamber at 28 °C under dark conditions for two days. After germination, seedlings with uniform size were transferred to new MS medium with or without OTC. To study whether OTC-induced arrest of root growth was related to a decline in H_2_O_2_ levels in the root tips, we added H_2_O_2_ at 1 mM to the growth medium to determine whether root growth could be recovered. In addition, we added 0.2 μM DPI, an inhibitor of H_2_O_2_ production, to the growth medium to determine whether the effects of DPI and OTC on root growth were comparable. To determine the role of NO in OTC-induced arrest of root growth, 250 μM cPTIO (the scavenger of NO), 100 μM tungstate (the inhibitor of nitrate reductase), and 25, 50, 100 or 200 μM L-NAME (an inhibitor of NO synthase activity) were added to the medium. The seedlings were allowed to grow on vertically placed 13 cm × 13 cm petri plates in a growth chamber at 25 °C under a 12 h photoperiod and photosynthetic photon flux density (PPFD) of 250 μmol m^−2 ^s^−1^.

### Analysis of primary root growth and microscopy

Soon after transfer of tomato seedlings to new MS media with or without OTC, the distal ends of the primary root tips were marked on the petri plates. After 3 d culture in medium, seedling phenotypes were photographed by scanning the petri plates. Primary root growth was evaluated by analysis of images taken from plates using ImageJ software (National Institutes of Health, US).

For analysis of meristem size, primary roots were fixed in ethanol/acetic acid (3:1, v/v) for 24 h and mounted in saturated chloral hydrate solution containing 10% glycerol. Measurements of root meristem size were performed with a microscope (Leica Microsystems, Wetzlar, Germany) using a 10x ocular and 5x objective. Meristem size was measured as the distance from the quiescent centers to the region where cells started to progressively increase in length as the result of rapid elongation.

### Determination of H_2_O_2_ level in the root tips

H_2_O_2_ in the root tips was detected with DAB and a H_2_DCF-DA fluorescent probe. For DAB staining, seedlings were immersed in buffer containing 0.1% DAB, 50 mM Tris-HCl, pH 3.8, at 25 °C for 30–60 min, and then the seedlings were washed three times with 50 mM sodium phosphate buffer, pH 7.4. After washing, 2 mm of root apex was excised and observed using a microscope (Leica Microsystems, Wetzlar, Germany). For H_2_DCF-DA staining, roots were incubated with 25 μM H_2_DCF-DA for 15 min and then washed three times with sodium phosphate buffer to remove excess fluorescent probe. After washing, 2 mm of root apex was excised and observed under a microscope with fluorescence detection unit (Leica Microsystems, Wetzlar, Germany) using excitation and emission wavelengths of 488 nm and 515 nm, respectively. The pixel intensities of fluorescence images were determined by Image J-software (NIH, Bethesda, MD, USA). Values were corrected for background.

### Determination of NO levels in the root tips

The accumulation of NO in the root tips was visualized using an NO-specific fluorescent probe, DAF-FM DA. The roots of seedlings were incubated with 5 μM DAF-FM DA for 1 h and washed three times with 50 mM sodium phosphate buffer, pH 7.4, to remove excess fluorescent probe. After washing, 2 mm of root apex was excised and observed through Laser Scanning Confocal Microscopy (LSCM-500, Zeiss, Germany) with excitation and emission wavelengths of 488 nm and 515 nm, respectively. The pixel intensities of fluorescence images were determined by using Image J-software (NIH, Bethesda, MD, USA). Values were corrected for background.

### Flow cytometry analysis

Cell cycle analysis was carried out by using flow cytometry. Samples, which contained at least 50 root tips each (2 mm), were chopped in 1 mL ice-cold buffer (45 mM MgCl_2_, 30 mM sodium citrate, 20 mM MOPS, pH7.0, and 0.1% Triton X-100) with a razor blade, filtered through 45-μm nylon mesh and stained with 200 μL PI/RNase Staining Buffer (Becton, Dickinson and Company, USA) for 20 min. Samples were analyzed with a FACS Calibur instrument (Becton, Dickinson and Company, USA). Cell cycle distribution was calculated using ModFit software (Verity Software House, US).

### Gene expression analysis

For analysis of the temporal expression pattern of cell cycle genes, germinated seeds were transferred to MS medium containing 2 mM HU, as described by Cools *et al*.^19^ OTC was added to the medium to study its effects on cell cycle genes. Samples of at least 30 root tips of 2 mm in length were harvested for gene expression. Total RNA was extracted by using an RNAsimple Total RNA Kit (Tiangen, DP419, Beijing, China) according to the manufacturer’s instructions. Total RNA (1 μg) was used to reverse-transcribe the cDNA template with a ReverTra Ace qPCR RT Kit (Toyobo, FSQ-301, Osaka, Japan).

The qRT-PCR assays were performed using a LightCycler^®^480 II Real-Time PCR detection system (Roche, Swiss). PCR was performed using SYBR Green PCR Master Mix (Takara, RR420A, Japan). The PCR conditions consisted of denaturation at 95 °C for 3 min, followed by 40 cycles of denaturation at 95 °C for 15 s, annealing at 58 °C for 15 s and extension at 72 °C for 30 s. The tomato *actin* gene was used as an internal control. Gene-specific primers were designed according to cDNA sequences, as described in Supplemental Information. Relative gene expression was calculated as described by Livak and Schmittgen^40^.

### Statistical analysis

The data were subjected to statistical analysis by ANOVA using SPSS for Windows version 18.0 (CoHort Software, Berkeley, CA, USA). Means were compared with Student’s t test or Tukey’s test at a significance level of *P* < 0.05.

## Additional Information

**How to cite this article:** Yu, Q.-X. *et al*. Nitric oxide is involved in the oxytetracycline-induced suppression of root growth through inhibiting hydrogen peroxide accumulation in the root meristem. *Sci. Rep.* **7**, 43096; doi: 10.1038/srep43096 (2017).

**Publisher's note:** Springer Nature remains neutral with regard to jurisdictional claims in published maps and institutional affiliations.

## Supplementary Material

Supplemental Information
