# Supplementary material for: Nitric oxide is involved in the oxytetracycline-induced suppression of root growth through inhibiting hydrogen peroxide accumulation in the root meristem
Source: Sci Rep. 2017 Feb 21;7:43096. doi: 10.1038/srep43096 (PMC5318916; doi:10.1038/srep43096)
Supplement: Supplemental Information [file srep43096-s1.doc]

**Nitric oxide is involved in the oxytetracycline-induced suppression of root growth through inhibiting hydrogen peroxide accumulation in the root meristem**

Qing-Xiang Yu^a^, Golam Jalal Ahammed^a^, Yan-Hong Zhou^a^, Kai Shi^a^, Jie Zhou^a^, Yunlong Yu^c^, Jing-Quan Yu^a,b^, Xiao-Jian Xia^a,*^

**Figure S1**

**A**

**B**

**Figure S1.** Effects of treatment with different concentrations of oxytetracycline (OTC) on primary root growth (A) and lateral root number (B) in tomato seedlings. Data represent mean ± SE (n=15, **P* value < 0.05, two-sided Student’s t test).

**Figure S2**

**Figure S2.** Effects of treatment with different concentrations of L-NAME on amelioration of inhibition of root growth by oxytetracycline (OTC) in tomato seedlings. Data represent mean ± SE (n=15, **P* value < 0.05, two-sided Student’s t test).

| **Supplemental Table S1 Primers used for qRT-PCR** | | | |
| --- | --- | --- | --- |
| **Genes** | **Accession numbers** | **Forward primer****s (5’-3’)** | **Reverse primers (5’-3’)** |
| *CycA3;2* | Solyc04g078310 | ACGAATGTTATCACCAGGCA | GGAGCGAGTTTAGCACATCA |
| *CycD3;3* | Solyc04g078470 | ACCAGGGAGTCCAACAGG | ATGGCTCAGGTGATGAAGTA |
| *CycB1;1* | Solyc10g078330 | ATTGCAGTTGTTGGGCATTA | CACGTAAGGTGTTGGGACTG |
| *CycB1;2* | Solyc10g080950 | AAGAAATTTGGGCACCTGAG | ACAGCAGCTTTGATGAATCG |
| *CycD3;1* | Solyc01g107730 | TCCCAGTAGTCCAAGTGGTG | TTTGCTGTTCTTGAACTTTGC |
| *CDKB2;1* | Solyc04g082840 | CACAGCAGTTGACATGTGGT | CCAGGCCAGAGTTCTTCATT |
| *NR* | Solyc11g013810 | ATTTGGAACGTCATGGGAAT | GCCTTATCAGAATGAATTGC |
